# Aligned Bioelectronic Polypyrrole/Collagen Constructs for Peripheral Nerve Interfacing

**DOI:** 10.1002/adem.202301488

**Published:** 2024-01-24

**Authors:** Ryan P. Trueman, Owein Guillemot-Legris, Henry T. Lancashire, Abijeet S. Mehta, Joshua Tropp, Rachel E. Daso, Jonathan Rivnay, Alethea B. Tabor, James B. Phillips, Bob C. Schroeder

**Affiliations:** UCL Centre for Nerve Engineering, University College London, London WC1N 1AX, UK; Department of Pharmacology, UCL School of Pharmacy, University College London, London WC1N 1AX, UK; UCL Centre for Nerve Engineering, University College London, London WC1N 1AX, UK, Department of Pharmacology, UCL School of Pharmacy, University College London, London WC1N 1AX, UK; Department of Medical Physics and Biomedical Engineering, University College London, London WC1E 6BT, UK; Department of Biomedical Engineering, Northwestern University, Evanston, IL 60208, USA; Department of Biomedical Engineering, Northwestern University, Evanston, IL 60208, USA; Department of Biomedical Engineering, Northwestern University, Evanston, IL 60208, USA; Department of Biomedical Engineering, Northwestern University, Evanston, IL 60208, USA; Department of Chemistry, University College London, London WC1H 0AJ, UK; UCL Centre for Nerve Engineering, University College London, London WC1N 1AX, UK, Department of Pharmacology, UCL School of Pharmacy, University College London, London WC1N 1AX, UK; Department of Chemistry, University College London, London WC1H 0AJ, UK

**Keywords:** bioelectronics, nerve repair, neural engineering, polypyrrole, tissue engineering

## Abstract

Electrical stimulation has shown promise in clinical studies to treat nerve injuries. This work is aimed to create an aligned bioelectronic construct that can be used to bridge a nerve gap, directly interfacing with the damaged nerve tissue to provide growth support. The conductive three-dimensional bioelectronic scaffolds described herein are composite materials, comprised of conductive polypyrrole (PPy) nanoparticles embedded in an aligned collagen hydrogel. The bioelectronic constructs are seeded with dorsal root ganglion derived primary rat neurons and electrically stimulated in vitro. The PPy loaded constructs support a 1.7-fold increase in neurite length in comparison to control collagen constructs. Furthermore, upon electrical stimulation of the PPy-collagen construct, a 1.8-fold increase in neurite length is shown. This work illustrates the potential of bioelectronic constructs in neural tissue engineering and lays the groundwork for the development of novel bioelectronic materials for neural interfacing applications.

## 1 Introduction

Peripheral nerve tissue has the innate ability to regenerate following traumatic injury. A cascade of molecular events occur that clear the injury site of cellular debris, reprogram endogenous Schwann cells and macrophages to be pro-regenerative, with these cells aiding nerve regeneration. However, this process is not always sufficient in cases of severe injuries that cause large gaps within the nervous system.^[1]^ In order to assist the regenerating neurons across the gap, surgical options include implantation of nerve autografts or nerve guidance conduits.^[2]^

The autograft is the current gold standard for repairing gaps within the peripheral nervous system caused through trauma.^[3, 4]^ However, this results in dissatisfactory functional recovery in 50% of patients,^[5]^ as well as requiring the harvest of limited donor peripheral nerve tissue.^[6]^ There has been extensive research and development into biomaterials that can be used to bridge the gap and which aim to surpass and replace the limited therapeutic potential of the autograft. Options that are currently available for use in the clinic are tubes with hollow lumen or decellularized allograft tissue.^[2]^ However, these approaches lack the living Schwann cells and other features of autograft tissue, which remains the current standard of care.

Recent research trends within the field of peripheral nerve tissue engineering have investigated the use of aligned biomaterials,^[7-10]^ conductive materials,^[11-15]^ and external electrical stimulation^[16-20]^ to accelerate regenerative outcomes. By aligning features within a biomaterial, the regenerating nerve is provided with guidance which can aid functional recovery.^[21]^ Bioelectricity is emerging as another beneficial tool within tissue engineering,^[11]^ with conductive materials offering attractive surface chemistry, the ability to conduct ionic and electronic charges and act as extracellular matrix scaffolds for regenerating cells.^[13, 22-25]^ These conductive materials can alter Schwann cell proliferation, adhesion, and phenotype, both as a passive, nonelectrically stimulated material, and also when charge is passed through the material.^[11, 26]^ Electrical stimulation through a polypyrrole (PPy)/chitosan biomaterial enhanced the expression of brain-derived neurotrophic factor and nerve growth factor in Schwann cells in vitro,^[24]^ highlighting the benefits of providing active stimulation through conductive materials. Conductive composites derived from collagen with incorporated poly(3,4-ethylenedioxythiophene) polystyrene sulfonate (PEDOT:PSS) and polyaniline nanofibers supported both PC-12 neuronal cell proliferation and differentiation, promoting expression of two neuronal differentiation markers (microtubule associated protein 2 (MAP2), β-III tubulin) and neurite extension in vitro without the application of electrical stimulation.^[23]^

Electrical stimulation, when applied to regenerating peripheral nerves, can increase the speed of peripheral nerve regeneration following surgical repair.^[16, 18, 27]^ By providing brief electrical stimulation of 1 h at the time of repair, it is possible to improve motor and sensory nerve regeneration in acute and chronic animal models of nerve injury.^[16]^ This increase in regeneration is hypothesized to be due to increased cyclic AMP within neuronal cell bodies, and an induction of rapid and sustained upregulation of regeneration associated genes, which in turn increases axonal outgrowth speed.^[27, 28]^

Therefore, we have sought to combine the benefits of biomaterial alignment with organic semiconducting nanoparticles to fabricate bioelectronic, tissue-engineered constructs. Gel-aspiration-ejection (GAE), a technique for rapidly manufacturing aligned collagen constructs, was used to create bioelectronic composites with aligned features and mechanical properties that can match endoneurial nerve tissue.^[29]^ A novel electrical stimulation platform was developed and characterized and then used to stimulate primary rat neurons within constructs featuring different concentrations of conductive filler. Characterization was performed to help investigate the relationships between construct fabrication, conductivity, electrical stimulation, and cell behavior, to aid future bioelectronic construct design.

## 2 Results and Discussion

### 2.1 Synthesis and Characterization of PPy Nanoparticles

**Figure 1.**
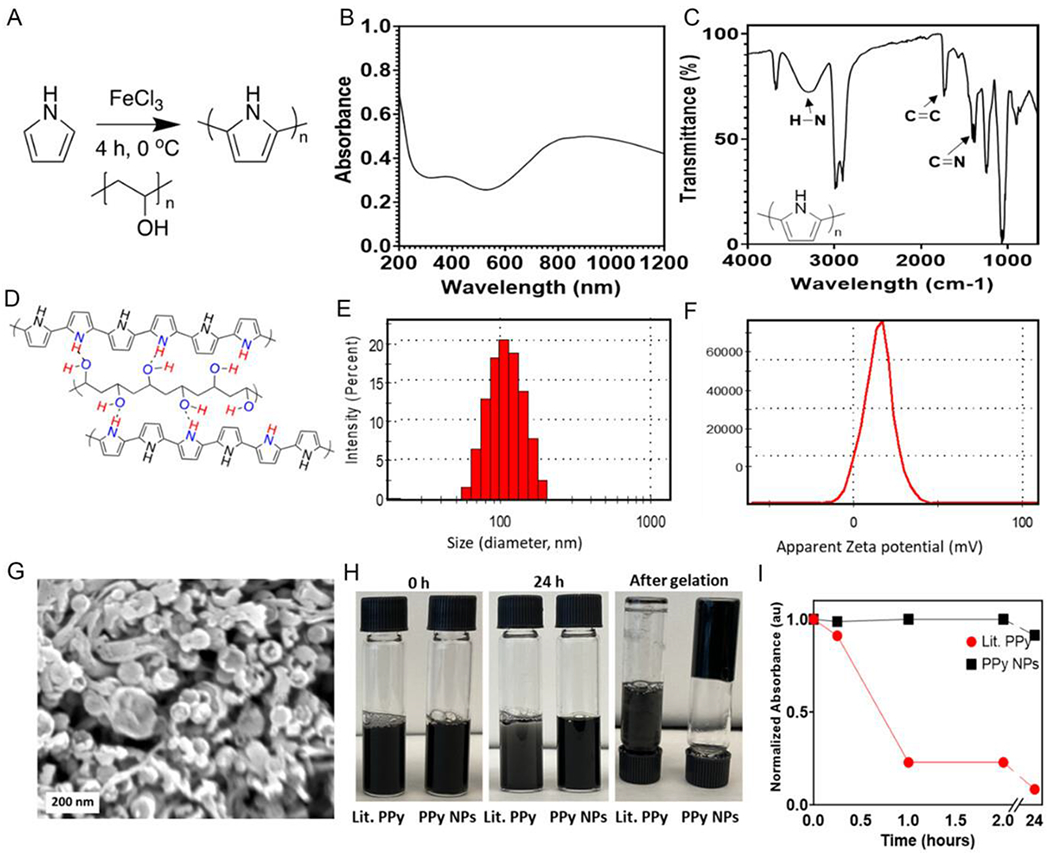
Synthesis and characterization of polypyrrole nanoparticles A) Scheme of the PPy nanoparticle (NP) synthesis, using PVA as a templating reagent with FeCl_3_ as the oxidative agent within the polymerization. B) UV/Vis spectrum of a dispersion of the PPy nanoparticles between 200 and 1200 nm (100 μg mL^−1^). C) FTIR spectrum of the PPy nanoparticles. D) Schematic showing the hypothesized arrangement of the PPy around the PVA based upon the hydrogen bonding between the two polymers. Hydrogen bonding is represented with dashed lines. E) DLS sizing of the synthesized PPy nanoparticles, with frequency of distribution. (*n* = 3) F) Zeta potential distribution of the PPy nanoparticles. (*n* = 3) G) SEM of the PPy nanoparticles. Scale bar = 200 nm. H) Photographs showing the experimental dispersibility of (left) PPy nanoparticles previously reported^[35]^ (Lit. PPy), and (right) the as synthesized PPy nanoparticles with PVA as a templating agent (PPy NPs). Photographs are immediately after agitation, 24 h later and after gelation using 1 m NaOH. I) UV/Vis analysis of PPy content within the meniscus of the prepared collagen/PPy solutions for Lit. PPy and the PPy NPs. Absorbance was normalized to the primary absorption feature of PPy, and later time points were compared against the starting value to understand the presence of PPy over time.

PPy was synthesized through a previously reported low-temperature templated oxidative polymerization using low-molecular-weight poly(vinyl alcohol) (PVA) as the templating polymer to generate nanoparticle structures and using ferric chloride (FeCl_3_) as the oxidant (Figure 1A).^[30]^ UV-vis absorption measurements show distinctive features typical of PPy.^[31]^ Previously reported PPy films showed a lower magnitude absorbance peak around 800 nm,^[32]^ whereas the PPy-PVA dispersion was found to absorb more strongly in this region in comparison (Figure 1B). Fourier transform infrared (FTIR) analysis was used to confirm the chemical composition and showed the presence of typical vibration bands associated with PPy; N-H (3600 cm^−1^), C = C (1540 cm^−1^), and aromatic C = N (1470 cm^−1^) (Figure 1C). After purification via dialysis in a 200 fold excess of double deionized H_2_O (ddH_2_O), the C—H peaks (2900 to 3000 cm^−1^) and O—H peak (3200 to 3400 cm^−1^ broad) originating from PVA were still present, highlighting the strength of the interaction between the PVA and the PPy within the nanoparticle structure.^[33]^ Figure 1D represents a schematic of the potential arrangement of the PPy and the PVA respective to the hydrogen bonding groups present within both the PVA and the PPy.^[34]^

The size distribution of the nanoparticles was determined by dynamic light scattering (DLS) (Figure 1E). The templating polymer facilitated the formation of narrowly distributed nanoparticles, with an average diameter size of 102 nm (0.08 polydispersity index (PDI)). The zeta potential of the nanoparticles was measured to be 14.7 ± 8.8 mV and allows the formation of homogenously dispersed solutions, preventing excessive particle aggregation during the collagen/PPy composite scaffold fabrication (Figure 1H).

The morphology of the PPy nanoparticles was further investigated by scanning electron microscopy (SEM), shown in Figure 1G, highlighting the spherical nature of the particles. However, there appear to be ribbon like structures present, which are most likely residual PVA left after purification. The presence of PVA after purification is also supported by ^1^H NMR measurements of the nanoparticles suspended in D_2_O (Figure S1, Supporting Information). ^1^H NMR spectra show the OH signals at 4.8, CH at 4.0, and the CH_2_ peaks between 2.0 and 1.5 ppm. The FTIR spectra also still possess the C—H peaks (2900–3000 cm^−1^) and O—H peak (3200–3400 cm^−1^ broad) from the PVA (Figure S2, Supporting Information). To gain further insight into the behavior of PPy nanoparticles, previously reported PPy nanoparticles that did not possess PVA as a templating agent were synthesized (Lit. PPy).^[35]^ Dispersibility of the nanoparticles was experimentally determined by allowing a solution of collagen with Lit. PPy particles and the PPy/PVA nanoparticles at 5% w/w of PPy in relation to collagen, to sediment within a collagen solution (Figure 1H).^[36]^ During the experiment, 15 μL samples were taken from the meniscus of the solutions, and UV/Vis measurements performed. The absorption features at 800 nm of the PPy dispersions were then used to quantify the amount of PPy present at the top surface of the collagen solution in comparison to the first time point (Figure 1I). The PPy nanoparticles possessed excellent dispersibility characteristics, with the particles remaining completely dispersed over 2 h, and furthermore the solution successfully set as a hydrogel after the collagen solution was neutralized. The Lit. PPy did not remain dispersed over the 2-hour experiment and impaired collagen gelation, failing to produce a robust hydrogel (Figure S3, Supporting Information).

Rheological studies indicated that the addition of the PPy nanoparticles to the collagen gels did not alter the storage (G′) and loss (G″) moduli of collagen, and in all materials, the G′ of and G′′ changed over the entire frequency range (0.1–10 Hz), indicating viscoelastic properties (Figure S4, Supporting Information).

### 2.2 Fabrication of Bioelectronic Constructs

Conductive properties have been shown to improve cellular interactions within neural tissue engineering, primarily through improved cell adhesion and phenotype alterations.^[22, 37]^ To this end, tissue-engineered constructs with PPy nanoparticles as electroactive filler were fabricated that possessed three potentially beneficial features^[2]^ such as alignment,^[9, 38]^ soft mechanical propeties,^[39, 40]^ and altered electrical features and conductive properties.

**Figure 2.**
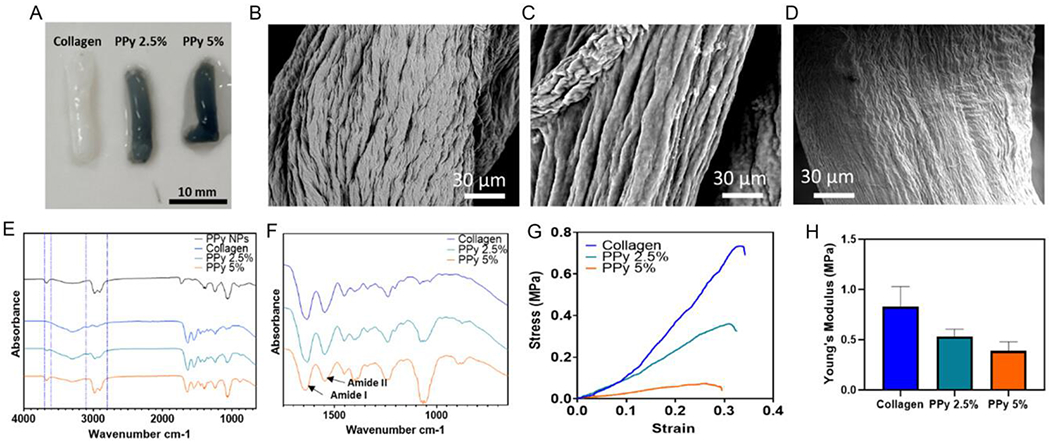
Fabrication and characterization of conductive scaffolds A) Photograph of the bioelectronic constructs with an unmodified collagen construct for control. B–D) Scanning electron microscopy of the surfaces of the constructs for collagen, PPy 2.5% and 5%, respectively. E) FTIR analysis of lyophilized constructs. The absorbance features of the PPy NPs were used to qualitatively determine incorporation of the nanoparticles into the constructs at 3000 and 3600. F) FTIR analysis of the amide region of the constructs. G) Representative tensile mechanical analysis of the constructs, pulled to failure. H) Young’s modulus, calculated from the linear region of the stress–strain curve, using *n* = 3 constructs. Data presented as mean +/− Std. Dev.

Previous literature interfacing PPy with biological systems has primarily focused on using PPy thin films^[41-43]^; however, to translate organic semiconducting polymers toward tissue engineering applications, 3D structures are required.^[44]^ PPy thin films are able to support PC-12 neurite extension, and this is enhanced when an brief electrical stimulus is passed through the PPy thin films,^[43]^ providing rationale to include PPy as a electroactive material. Through the addition of two different concentrations of PPy nanoparticles (2.5% and 5% w/w) into a fibrillar collagen gel, it was possible to produce collagen-based constructs (Figure 2).

The GAE technique provides anisotropy to the hydrogel and removes the bulk volume of water.^[29]^ Three different constructs with two concentrations of PPy nanoparticles were fabricated (Figure 2A). The bioelectronic constructs embedded with the PPy nanoparticles became increasingly black in color as the concentration of PPy nanoparticles increased.

The surface morphology of the bioelectronic constructs was imaged by SEM and revealed texture in the longitudinal axis consistent with alignment produced through the GAE method (Figure 2B–D).^[45]^ The aligned texture was less prominent in the sample with the greater loading of PPy nanoparticles.

To verify PPy nanoparticle encapsulation within the bioelectronic constructs, FTIR was used (Figure 2E). FTIR of the collagen and collagen/PPy constructs displays an increased magnitude of the N—H peak (3600 cm^−1^) from the PPy and the C—H peak (3000–2900 cm^−1^) from the templating PVA within the PPy 2.5% sample. These peaks were further increased in the PPy 5% construct, indicted on the spectra with blue dashed lines. This confirms the successful incorporation of the PPy nanoparticles into the bulk of the collagen and the ability for PPy nanoparticles to be retained within the constructs after washing in excess ddH_2_O overnight. FTIR absorbance features from amide I and II features can be analyzed to give insight into the collagen fibrillization,^[46, 47]^ and if this process has been disrupted at all by the addition of the conductive filler within the final constructs (Figure 2F). There appears to be little difference between the collagen and PPy 2.5% constructs, with both exhibiting a maximum peak absorbance of 1633 and 1547 cm^−1^ for amide I and II, respectively. However, within the collagen construct with the higher loading of PPy nanoparticles, the peak maximum absorption was slightly shifted to 1645 and 1551 cm^−1^, suggesting that there are differences within the collagen fibrillization between the composite material and collagen alone. A recent study highlighted that changes in this region of the FTIR spectra can be indicative of when the collagen fibrils are denatured,^[47]^ with this finding suggesting that the PPy nanoparticles are slightly disrupting the collagen fibrillization within the constructs as evidenced by the shift of this absorption feature in Figure 2F.

One of the issues commonly faced with bioelectronic material development is that addition of conducting component makes the material stiffer, creating mechanical mismatch at the bioelectronic to tissue interface. However, organic, carbon-based electronics circumvent this issue as they are softer than their inorganic counterparts.^[48, 49]^ Mechanical analysis using a tensile pull to failure test (Figure 2G) highlighted that as PPy nanoparticle concentrations increased, Young’s modulus, and ultimate tensile stress (Figure 2H) were reduced. At lower strains of below 1%, the collagen and PPy 2.5% exhibited similar stiffness. Ultimate strain was similar between the constructs, indicating the materials could be stretched to a similar extent before breaking (Figure S5, Supporting Information).

Small-angle X-ray scattering (SAXS) performed using a 0.5 mm beam width (Figure S6, Supporting Information) revealed that the collagen sample was anisotropic, whereas this anisotropy was slightly disrupted in the samples containing PPy nanoparticles. The FTIR, DMA, and SAXS characterization together indicate that the addition of PPy slightly alters the collagen organization within the constructs, but that the overall structural features are predominately conserved.

### 2.3 Electrical Properties of the Bioelectronic Constructs

**Figure 3.**
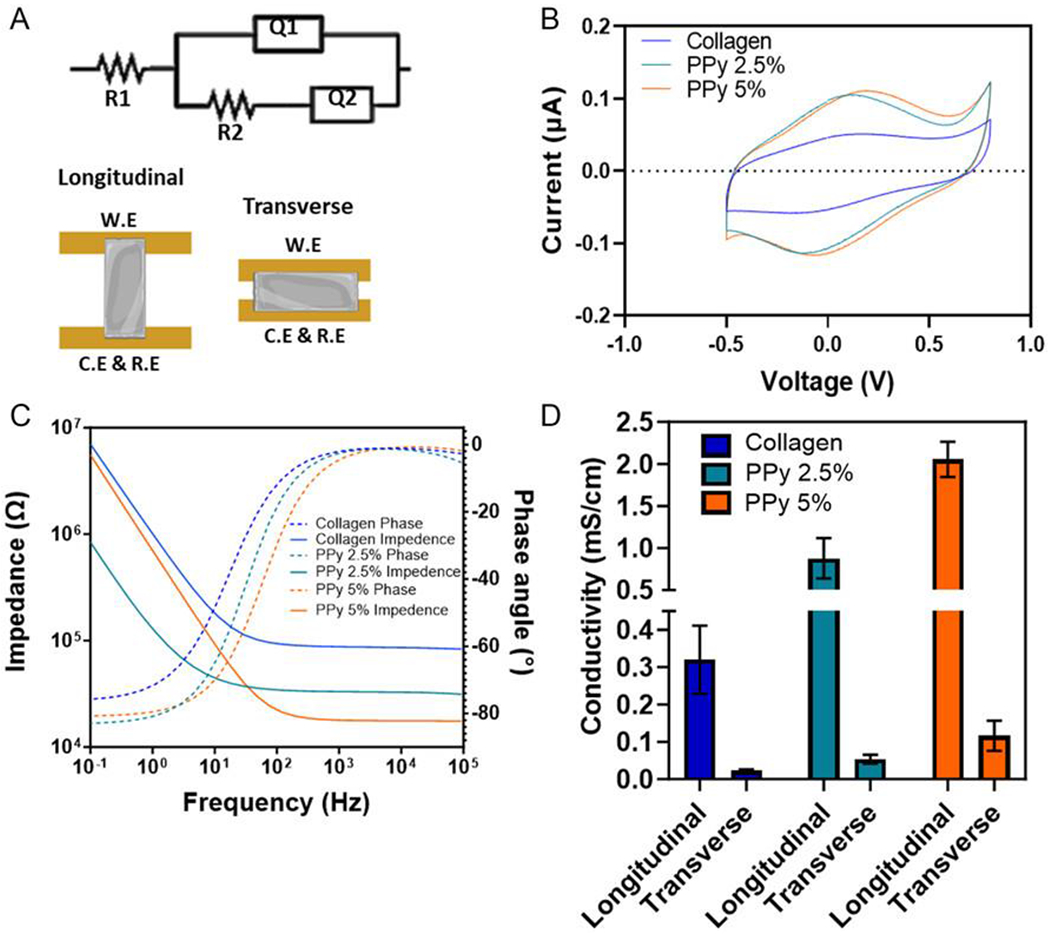
Electronic characterization of the bioelectronic constructs A) Schematic for the conductivity testing apparatus, with gold planar electrodes for both the working electrode (W.E.), counter and reference electrodes (C.E. and R.E.) used with the hydrated constructs contacting the electrodes. Circuit fitting diagram used for calculating the conductivity of the constructs, using the second resistor (R2) value of resistance to calculate the conductivity (Q1 and Q2 = capacitors 1 and 2, respectively). B) Cyclic voltammetry of the constructs to assess if the addition of PPy to the collagen altered the electrochemical properties of the material. C) Representative electrochemical impendence spectroscopy used within the circuit fitting equation to determine conductivity. D) Conductivity values of the constructs, both longitudinal and transverse, to evaluate the directionality of the electrical properties. *N* = 3, data presented as mean +/− Std. Dev.

Electrochemical impedance spectroscopy (EIS) combined with fitting representative circuits was used to perform cyclic voltammetry and determine the conductivity both parallel to the long axis of the bioelectronic constructs and across the width of the constructs, with a schematic highlighting the set up in Figure 3A and representative EIS spectra shown in Figure 3C. EIS of the constructs demonstrates that conductivity positively correlates with PPy nanoparticle content in both directions. However, measured conductivity was magnitudes greater along the long axis of the construct, the direction of GAE alignment, in comparison to the transverse conductivity, possibly due to the anisotropic structure within the construct.

Cyclic voltammetry was utilized to determine if the addition of the PPy nanoparticles altered the electrochemical properties of the bulk material (Figure 3B). Within the cyclic voltammogram, differences in the redox peaks are shown, with both constructs incorporating PPy nanoparticles having altered redox features in comparison to collagen, indicative of addition of electroactive components.

Through the addition of PPy nanoparticles, longitudinal conductivity of the constructs increased by over sixfold for the highest concentration of PPy in comparison to the collagen only construct, with 0.31 ± 0.07, 0.88 ± 0.19, and 2.06 ± 0.17 mS cm^−1^ for collagen, PPy 2.5%, and PPy 5%, respectively (Figure 3D); The same trend is true of the transverse conductivity measurements, with increasing amount of conductive filler resulting in increases in conductivity. However, the conductivity is considerably lower across the width of the constructs when comparing longitudinal conductivity to transverse conductivity, with the PPy 5% GAE construct possessing a mean conductivity of 2.05 mS cm^−1^ across the length of the construct and 0.17 mS cm^−1^ across the transverse section of the construct. Conductivity of the highly hydrated gels of each composite material was investigated before the application of GAE (Figure S7, Supporting Information). This revealed that through the process of GAE, the conductivity of the PPy containing materials increased, likely due to the relative increase in density of the PPy nanoparticles following GAE. In the case of PPy 5%, after GAE, the conductivity across the longitudinal axis increased 10-fold.

However, important to note is that construct conductivity is low compared with typical cell culture medium conductivity, which is approximately 14 mS cm^−1^. The exact mechanism responsible for the increased conductivity within the PPy containing constructs in comparison to collagen was not explored within this study; however, it is hypothesized that the mixed ionic/electronic conductivity of the construct described would explain the increase in conductivity.^[50, 51]^

Recently published work on aligned conductive 3D scaffolds for skeletal muscle engineering reported a similar trend between increasing concentration of PPy within their collagen scaffolds, with conductivities reported between 27 mS cm^−1^ for the collagen only scaffold, and 142 mS cm^−1^ for 0.5% PPy inclusion.^[35]^ The manufacturing methods between this study and the cited study make it difficult to directly compare total amounts of PPy and their relative amount of electroactive filler. However, the trend of increasing PPy content within the collagen constructs results in increased conductivity of the bulk material matches our observations.

Our study, however, highlights that when considering complex architectures, regularly encountered in tissue engineered structures, increasing the conductivity using electroactive fillers can alter other properties of the construct, so detailed characterization should be considered during bioelectronic construct development.

### 2.4 Design of a Novel Electrical Stimulation Platform for Use with Fabricated Constructs In Vitro

**Figure 4.**
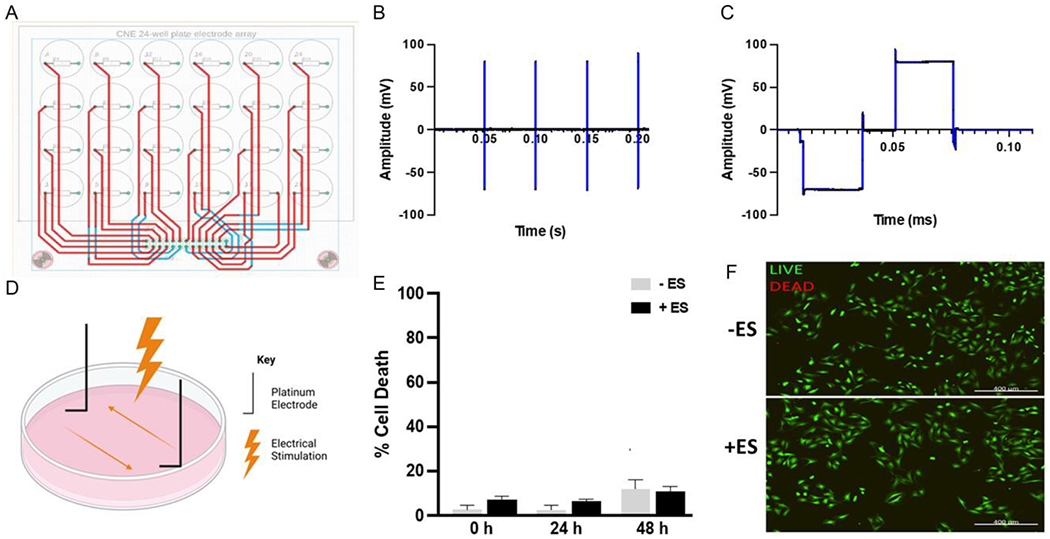
Design and evaluation of an electrical stimulation platform for construct stimulation. A) Autodesk eagle schematic of the CNEStimulator printed circuit board (PCB) based lid for interfacing with a standard cell culture 24-well plate. B) 20 Hz pulse train generated by the CNEStimulator in saline solution. C) Biphasic, charge balanced, cathodic first 300 μA, 200 μs per phase pulse voltage waveform during stimulation in saline solution. D) Graphic displaying how the electrodes deliver electrical stimulation to cell culture media in vitro. E) LDH assay of media from a monolayer culture of SCL4.1/F7 Schwann cells, taken at 3 separate time points after electrical stimulation. Stats = 2-way ANOVA, with no significance detected. F) Fluorescence micrograph of SCL4.1/F7 Schwann cell after 48 h post electrical stimulation. Live (green): calcein-AM, Dead (red): ethidium homodimer-1. Scale bar = 400 μm.

A device was developed to enable controlled electrical stimulation to be applied to specific cell populations in vitro. Once developed, the waveform and magnitude of the electrical stimulation were characterized. Cell viability was evaluated using a rat Schwann cell line, SCL 4.1/F7, as a relevant cell type involved with peripheral nerve regeneration (Figure 4).

The Centre for Nerve Engineering Stimulator (CNEStimulator) consists of an Arduino-based voltage waveform generator, a 10-channel voltage-to-current converter, and a printed circuit board (PCB) “lid” and electrodes to interface with traditional cell culture plasticware (Figure 4A). The PCB is silicone encapsulated to reduce corrosion risk during operation in the high humidity cell culture incubator.

There are different systems reported for delivering various electrical stimulation parameters, e.g., alternating current (AC)^[52]^ vs direct current^[25]^ (DC). By using AC within this study, it is possible to leverage current knowledge of ES within peripheral nerve repair,^[27]^ while avoiding the issues of DC systems, including the generation of cytotoxic pH gradients across the potential difference field and at electrode interfaces.^[53]^

The CNEStimulator features L-shaped platinum electrodes to minimize corrosion^[54]^ during operation and deliver AC pulses with low-voltage transient amplitude and gradient (20 Hz, 60 mV cm^−1^) (Figure 4B,C) to prevent redox reactions and ion gradients across the cell culture media. One drawback to the present system is the slight overshoot in current at the start of the positive portion of the wave.

The stimulation parameters used here were based on previous preclinical studies showing improved motor axon regeneration in a rat femoral nerve transection model,^[55]^ associated with upregulation of regeneration associated genes in neurons^[27]^ and are currently being investigated clinically for the treatment of chronic compression nerve injuries.^[16]^

Before investigating the effect of electrical stimulation on neurons growing within the constructs, cell compatibility of the CNEStimulator was assessed through electrical stimulation of SCL 4.1/F7 rat Schwann cells seeded within a 24-well plate and stimulated at 20 Hz for 1 h. The lactate dehydrogenase (LDH) assay showed similarly low levels of cell death under electrical stimulation within each sample 48 h after electrical stimulation, with both the control and the electrically stimulated samples exhibiting ≈10% cell death. There was a trend toward slightly increased cell death in stimulated samples immediately after application (Figure 4E).

### 2.5 Assessment of Neurite Extension within the Bioelectronic Constructs

Before culturing primary rat neurons within the bioelectronic constructs, we wanted to assess cytocompatibility. Schwann cells were cultured within the constructs, and LDH assay used to determine cell death (Figure S8, Supporting Information). The constructs at the PPy concentrations used did not cause any cell death over 10%, and hence the constructs were deemed suitable for the neurite extension assay.

**Figure 5.**
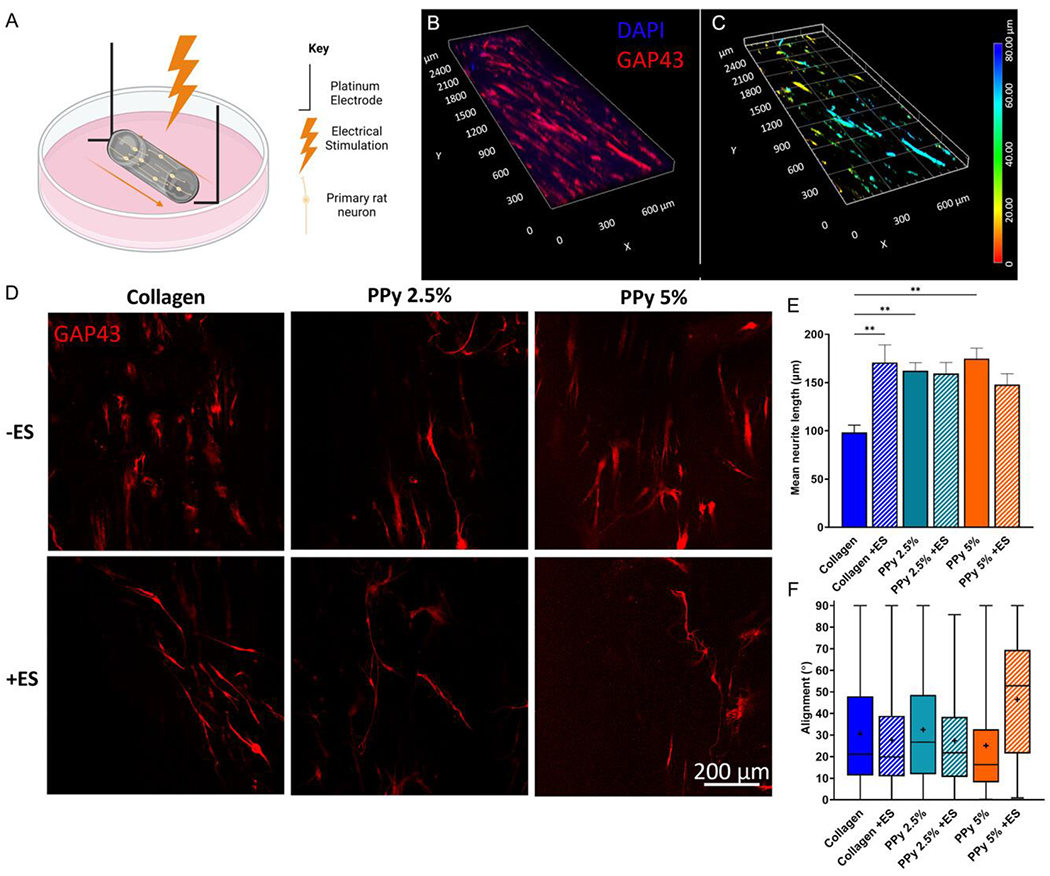
Neurite extension within the bioelectronic constructs under electrical stimulation. DRG neurons were incorporated within constructs and maintained in culture for 72 h. A) Schematic highlighting the placement and experimental setup of the electrically stimulated constructs. B) Representative confocal z-stack micrograph highlighting the 3D nature of the constructs and cellular growth within them. Sample is PPy 2.5%—ES. C) Representative depth microscopy taken from the confocal micrograph, highlighting the 3D nature and the differing depths of neuron placement and growth within the constructs. D) Representative single slices of confocal immunofluorescence micrographs with immunostaining of GAP-43 in neurons within the constructs. E) Quantification of mean ± SEM neurite length within the constructs, with (dashed) and without (solid) electrical stimulation. Statistics utilized two-way analysis of variance (ANOVA) and Šídák’s multiple variables test, *p < 0.05 and **p < 0.01. F) Box and whisker plots displaying the upper and lower interquartile ranges (25% and 75%, respectively), median (solid line), mean (+), and the min/max range of the alignment within the constructs. *n* = 6 independent constructs.

To investigate the effect of the material and of electrical stimulation on neurite outgrowth in vitro, dorsal root ganglion (DRG) neurons were cultured within the constructs (Figure 5A), with neurite extension occurring throughout the material in three dimensions (Figure 5B).

Figure 5C displays representative confocal micrographs showing GAP-43-positive neurons within the constructs after 72 h, with or without electrical stimulation. Figure 5D shows the neurite extension within the different constructs. Inclusion of electroactive filler PPy increased mean neurite length significantly (PPy 2.5% 1.6-fold increase, *p* = 0.0109, PPy 5% 1.8-fold increase, *p* = 0.0163, compared with collagen alone). Additionally, electrical stimulation caused a 1.7-fold significant increase in mean neurite length (*p* = 0.0107) within the collagen construct compared with the unstimulated control. Within the samples containing 2.5% and 5% PPy, there was no further increase in neurite length associated with electrical stimulation compared with the PPy-containing unstimulated controls (Figure 5D).

Inclusion of PPy nanoparticles in the constructs provided benefits to neurite growth support without electrical stimulation. It is interesting to note that providing electrical stimulation to the constructs containing PPy nanoparticles did not increase neurite extension any further than the elevated level detected due to the presence of the PPy. This lack of an additive effect may be a limitation of the experimental approach, reflecting perhaps a ceiling in terms of neurite extension rate in vitro, or it might indicate that electrical stimulation has little effect on neurite extension rate within PPy-loaded collagen.

These findings contrast with other studies using different approaches where electrical stimulation resulted in additional neurite growth. Modest increases to neurite extension on conductive substrates without application of an electrical field have been previously reported.^[56, 57]^ By culturing PC-12 neuronal cell line for 48 h on thick PPy films, a 1.2-fold increase in neurite length in comparison to tissue culture plastic was seen. However, in that study, when an electrical stimulus of 100 mV was passed through the PPy film for 2 h, neurite length is approximately twofold greater in comparison to the nonstimulated film.^[56]^ Additionally, DRGs encapsulated within collagen-type 1, single-walled carbon nanotube composite hydrogels presented an increase in neurite length in the absence of electrical stimulation compared to collagen-type 1 hydrogels. However, when a 50 mV mm^−1^ DC electrical stimulation for 8 h was applied to the conductive material, DRG neurite outgrowth was further increased.^[57]^ Within our study, materials are not connected directly to the power supply, but electrical stimulation is hypothesized to be created wirelessly within them by electrodes connected to the culture medium in which they are immersed, similar to previous work in the field.^[25]^

There is a need to understand the interplay between electrical stimulation, conductivity, and surface chemistry of biomaterials for tissue engineering and in vitro models can enable these factors to be investigated separately and in combination to help optimize the benefits in vivo^[11]^ and establish how different features may be synergistic or additive.

Alignment is an important feature within constructs intended for peripheral nerve repair, to help guide regeneration across the nerve gap into the distal stump. Within the manufactured constructs, neurite extension was predominantly aligned parallel to the long axis of the construct (Figure 5E). Collagen, PPy 2.5% and 5% without electrical stimulation yielded a mean alignment of 30.57° ± 24.32°, 32.53° ± 24.17°, and 25.14° ± 22.66° alignment, respectively, with 0° being parallel to the long axis. Electrical stimulation and electrical fields have previously been reported to influence directionality within neuronal cells.^[25, 58]^ Electrical stimulation did not alter the distribution of alignment within the collagen and PPy 2.5% constructs; however, in the PPy 5% sample, alignment was compromised by electrical stimulation, with a greater mean, median, and wider interquartile range in comparison to the other electrically stimulated constructs.

Previously, chick DRG neurons were cultured on the surface of aligned electrospun fibers, and electrically stimulated in a DC electrical field, and there was no influence of the electrical field on neurite directionality.^[59]^ This indicates that material alignment can be a more dominant feature than electrical stimulation in terms of cell guidance. Experimental work isolating the effect of electrical stimulation on neurite alignment on conductive materials without any aligning features used *X. laevis* spinal neurons stimulated for 3 h within a direct current electrical field at strengths of 50, 100, and 150 mV mm^−1^. In the highest strength electrical field of 150 mV mm^−1^, neurons exhibited a strong directionality bias toward the cathode and started growing directly toward the negative pole created within the culture.^[25]^

## 3 Conclusion

Novel PPy nanoparticle/collagen composite hydrogels were synthesized that possessed similar rheological profiles to collagen, which were subsequently processed into aligned constructs for neural tissue engineering through the GAE technique. Addition of conductive filler enhances conductivity within the collagen after GAE processing, whilst having minimal impact on the ability to form 3D constructs.

To evaluate the use of electrical stimulation in combination with the conductive bioelectronic constructs, a novel electrical stimulation platform was developed that allowed for direct charge injection into the cell culture wells in a reproducible manner. Primary neurons were incorporated within the constructs; neurite extension was increased through the addition of the PPy nanoparticles and through electrical stimulation in collagen-only constructs. This highlights both the passive benefits of conductive material and the active benefit of electrical stimulation to neurite extension in 3D.

## 4 Experimental Section

### Synthetic Reagents

Pyrrole was purchased from Fluorochem, UK, and prior to use, passed through an aluminium oxide (ThermoFisher, UK) plug. Polyvinyl alcohol (PVA) (9–10 kDa) was purchased from ThermoFisher and used without further purification. Anhydrous iron (III) chloride was purchased from Insight Biotechnology, UK. Slide-A-Lyzer dialysis cartridges (Pore size 10 000 Da) were purchased from ThermoFisher, UK.

### Polypyrrole Nanoparticle Synthesis

PPy nanoparticles were synthesized by a previously reported templated oxidative polymerization method.^[30]^ To a single necked 100 mL round-bottomed flask, 0.6 g (3.85 mmol) of anhydrous FeCl_3_ and low molecular weight PVA (1.5 g, 35.47 mmol) were added to 50 mL of deionized water at room temperature. This solution was stirred for 1 h to form a homogenous clear yellow solution. After this point, the solution was transferred to an ice water bath (4 °C) and left for 30 min for the reaction vessel to reach the lowered temperature. Pyrrole (0.14 g, 2.09 mmol) was added dropwise into the solution. After complete addition over 5 min, the reaction was continuously stirred for 4 h, before the reaction vessel was removed from the ice bath and allowed to reach room temperature. The resulting suspension was transferred to a dialysis cartridge and subjected to dialysis for 48 h in 1 L of double-deionized water to remove any excess FeCl_3_ and PVA, with 3 water changes over the 48-hour period, at 6, 12, and 24 h. After purification, the sample was freeze dried overnight to yield a fine, but slightly sticky black powder (0.11 g, 1.05 mmol, 50% yield). ^1^H NMR of the nanoparticles was recorded within D_2_O using a Bruker 400 Hz NMR instrument (S2, Supporting Information). PPy particles (Lit. PPy) from a previous experimental report were synthesized for dispersion comparison with FeCl_3_ under vigorous mixing for 24 h under ambient conditions.^[35]^ Lit. PPy was purified in the same manner as the PPy nanoparticles and used subsequently in Section 4.4.

### Characterization of Nanoparticle Size

The diameter and PDI of a 100 μg mL^−1^ suspension of PPy nanoparticles, prepared from freeze dried nanoparticles, were determined using a Zetasizer Nano ZS (Malvern Instruments). Size was determined using DLS and observing the Brownian motion of the particles. Nanoparticles were attached to a self-adhesive carbon disc and coated with 25 nm of gold using a sputter coater. The nanoparticles were imaged using a Phenom Pro benchtop SEM imaging at 10 kV accelerating voltage using secondary electron detection.

### Dispersibility of PPy Nanoparticles

Two preparations of 0.04 mg mL^−1^ PPy particles fully dispersed in 5 mL rat tail collagen I (2 mg mL^−1^ in 0.6% acetic acid; FirstLink, UK) were prepared in glass vials. The vials were then placed on a level surface to allow sedimentation, and 5 μL aliquots of the mixture were taken at various time points from the meniscus of the solution, and UV/Vis was performed using a NanoDrop One (ThermoFisher, USA). Data were normalized to the absorbance at the initial time point. After 24 h, the solutions were neutralized using NaOH in the same manner as described in Section 4.5.

### Fabrication of Bioelectronic Constructs

To prepare a solution of collagen gel, 800 μL of rat tail collagen I (2 mg mL^−1^ in 0.6% acetic acid; FirstLink, UK) was combined with 100 μL of 10X minimum essential media (MEM) with either no additional PPy nanoparticles, or 2 different concentrations of PPy nanoparticles. PPy nanoparticles were added to the 10X MEM, with 2.5% and 5% w/w of PPy nanoparticles in relation to the rat tail collagen (Table 1).

This mixture was neutralized on ice using dropwise additions of NaOH (0.925 and 0.185 m in double deionized (dd) H_2_O as determined by color change of phenol red indicator). After neutralization, 600 μL of collagen/PPy solutions were added to individual wells of a 48-well plate (well diameter = 0.8 mm, height of the gel = 0.6 mm). After the gels were added to the well plate, it was transferred to a cell culture incubator for 30 min (37 °C, humidified incubator, 5% CO_2_) to allow gelation.

After the gels had set, GAE was used to densify the gels and form the bioelectronic constructs^[29]^ GAE involves using an angioplasty device with an attached 16 G cannula to aspirate the gel, removing most of the interstitial water, resulting in approx. a 60-fold reduction in volume.^[29]^ Table 1 displays the final composition of the bioelectronic constructs.

### Rheological Characterization of the pre-GAE Collagen/PPy Hydrogels

Rheological measurements were carried out using a Bohlin Gemini Rheometer. 500 μL of hydrogel (before GAE) was placed between a 20 mm parallel plate with a gap size of 500 μm within a chamber to prevent excess moisture loss during measurements. Measurements were conducted at room temperature, between 20 and 25 °C. A frequency sweep from 0.1 to 10 Hz at a strain amplitude of 0.0005 was carried out on three independent hydrogels and the mean presented of the storage modulus (G′) and loss modulus (G″). Data were smoothed using the average of 5 neighboring points to the second-order smoothing polynomial and plotted using GraphPad Prism 7.

### Surface Morphology of Bioelectronic Constructs

Constructs were fixed overnight in paraformaldehyde (4% in phosphate-buffered saline (PBS)) and gradually dehydrated in increasing concentrations of ethanol/H_2_O, (50%, 60%, 70%, 80, 90% up to 100% ethanol), with two washes conducted in the 90% and 100% ethanol. Samples were removed from 100% ethanol, attached to a self-adhesive carbon disk (Taab, UK), completely soaked in hexamethyldisilane, and left to dry within a fume cupboard. The samples were sputter coated with 2 nm of gold and imaged using Phenom Pro benchtop SEM.

### Fourier Transform Infrared Spectroscopy

FTIR was used to acquire the IR spectra of the PPy nanoparticles before and after purification and the dried collagen and bioelectronic constructs in the 4000 to 600 cm^−1^ wavenumber range, with the average of 10 scans per spectrum taken, at a resolution of 2 datapoints per cm^−1^ (Perkin Elmer Spectrum 100).

### Tensile Mechanical Analysis of GAE Constructs

Quasi-static tensile testing of the constructs was performed using a Bose Electro Force (3200 Series II, TA Instruments) and WinTest 7 Software. Constructs were prepared to be at least 10 mm in length and approximately 1.2 mm in diameter (cannula gauge 16) and were placed between the instrument grips with a gauge length of 5 mm. All constructs were assumed to be cylindrical in shape. Samples were kept moist during testing by applying PBS to the constructs. Each specimen was stretched at a constant rate of 0.17 mm s^−1^ to complete tensile failure to obtain stress–strain relationship data. For all constructs, mean ultimate stress, mean ultimate strain, and Young’s modulus were determined from the initial length and diameter of the specimens and the force tracings measured during testing. Ultimate tensile stress refers to the amount of force per unit of initial cross-sectional area at tensile failure. Ultimate strain refers to the amount of elongation divided by the initial specimen length achieved at the point of tensile failure. Young’s modulus was calculated from the slope of the ascending linear portion of the stress–strain curve.

### Small-Angle X-ray Scattering of the Constructs

SAXS measurements were performed using the Rigaku SMAX 3000/Xenocs system at the J.B. Cohen X-ray diffraction facility at Northwestern University. X-rays (*λ* = 1.5418 Å, Cu Kα) from a Cu sealed tube source operating at 45 kV and tube current of 0.88 mA were condition by a microfocusing optic. Single crystal Si slits 0.5 × 0.5 mm^2^ place 5 cm before the sample defined the beam size at the sample position. The incident flux was ≈3 × 10^7^ photons s^−1^. The scattered intensity was collected in the transmission mode using a single photon counting, pixel array detector (Eiger R 1 m, pixel size: 75 × 75 μm^2^) placed 1.56 m away from the sample. The incident and the receiving flight paths were kept under vacuum to reduce air scattering background. The samples were mounted on Kapton (polyimide) tape and were centered in the beam using two translation stages normal to the beam direction. A total exposure time of 300 s was used per sample.

### Electrical Characterization of Fabricated Constructs

Electrochemical impedance spectroscopy (EIS) was used to assess the electrical properties of the constructs. EIS was performed on constructs after GAE had been performed and constructs soaked in deionized water overnight to remove the confounding effect of electrolyte on EIS. EIS was run at 31 frequencies between 0.1 Hz and 1 × 10^6^ Hz with a 10 mV_RMS_ AC amplitude. The resulting data were fit to an equivalent circuit with a resistor (R2) and capacitor (Q2) in series, in a parallel circuit with resistance from the circuit (R1) and capacitance of the electrolyte (Q1), excluding frequencies above 10^5^ Hz. The resistance (R) and capacitance (Q) values were extracted based on the circuit fits, and those numbers were averaged. Conductivity values were calculated based on the extracted resistance value (R2). All constructs were estimated to have a radius of 1.2 mm (determined from the cannular size of GAE formation (16G)) and were sliced into 5 mm long units to be placed between two gold electrodes 4 mm apart. To test the cross-sectional resistance, the constructs were placed between two gold electrodes 1 mm apart.

### Center for Nerve Engineering Stimulator (CNEStimulator) Development

A cell electrical stimulator was designed for in vitro current-controlled stimulation. The Center for Nerve Engineering well-plate stimulator comprised 3 parts. First, a voltage waveform generator was developed comprising an Arduino Uno R3 microcontroller, paired voltage dividers, TL072 dual operational amplifier buffers, and a DG403 dual SPDT analog switches to deliver the desired biphasic voltage waveform. A 10-channel voltage-to-current converter was used as previously described, comprising 10 parallel modified Howland current pumps assembled from INA103 instrumentation amplifiers.^[60]^ A custom cell culture lid was designed to fit 24-well cell culture plates, comprising a custom printed circuit board (PCBWay, China), with one pair of platinum wire electrodes per well (0.5 mm diameter, Advent Research Materials, UK). Solder joints between the PCB and platinum wire electrodes were protected with DOWSIL 734 silicone. A ribbon cable was used to connect the CNEStimulator cell culture lid to the multichannel voltage-to-current converter.

For all in vitro stimulation biphasic, current controlled, charge balanced, square pulses were used. Stimulation had 300 μA amplitude, 200 ms phase width, 50 ms interphase delay, equal phase amplitude and width, and stimulation was applied at 20 Hz. Therefore, 60 μC charge per phase was applied.

### CNEStimulator Characterization

The custom cell culture lid was placed over a 24-well cell culture plate. Each well was filled with 1 mL PBS to cover the electrodes to a depth of 4.9 mm, resulting in a stimulation electrode surface area of approximately 8 mm^2^. Biphasic current-controlled stimulation was applied as detailed in the stimulator development section, and the voltage transient response was measured between the stimulation electrode and the return electrode.

### 4.1 1/F7 Schwann Cell Viability Testing After Electrical Stimulation

SCL 4.1/F7 Schwann cells were procured from Sigma Aldrich and seeded within the bioelectronic constructs at a density of 500 000 cells per construct, for assessment of cell viability. Cells were added to the gel precursor after NaOH neutralization in 100 μL of Dulbecco’s modified Eagle medium, (DMEM) (ThermoFisher, UK), then cellular material underwent gelation followed by GAE stabilization. For assessment of the effect of electrical stimulation on Schwann cell viability, cells were seeded at a density of 15 000 cells per well in a 24-well plate, allowed to adhere for 24 h after seeding and electrically stimulated using the described parameters (Section 4.9) for 1 h, and then incubated for a further 48 h. Live/dead staining of SCL 4.1/F7 monolayer culture was performed according to the manufacturers protocol (ThermoFisher, UK), and images captured using an Incucyte S3. Second, the LDH assay (Abcam, UK) was conducted for the monolayer electrically stimulated culture according to the manufacturer’s protocol. Monolayer cultures and constructs seeded with Schwann cells were maintained in DMEM with 10% FBS, 1% Pen/Strep culture medium at 37 °C in a humidified incubator with 5% CO_2_.

### Culture of Neurons Within Constructs

All experimental procedures involving animals were conducted in accordance with the UK Animals (Scientific Procedures) Act (1986) and approved by the UCL Animal Welfare and Ethical Review Body. Dissociated DRG neurons were prepared from 3 adult (200–300 g) male Sprague Dawley rats culled humanely by CO_2_ asphyxiation according to local guidelines. DRGs were incubated in collagenase type IV (0.125%; Sigma) for 1.5 h at 37 °C then dissociated by trituration and washed three times with 20 mL of culture medium before being incubated for 48 h with cytosine arabinoside (0.01 mm) to deplete glia. The resulting cultures were seeded within the hydrogels during the gelation process, at a density of approximately 2 processed DRGs per construct, resulting in a sufficient number of neurons per construct. The constructs seeded with neurons were then processed using GAE and cultured in DMEM with 10% FBS, 1% Pen/Strep culture medium at 37 °C in a humidified incubator with 5% CO_2_. The seeded constructs were electrically stimulated for 1 h. After a further 72 h incubation post electrical stimulation, the constructs were washed briefly in PBS and fixed in 4% paraformaldehyde at 4 °C for 24 h, then immunofluorescence staining was carried out.

### Immunostaining of the Primary Neurons

To identify the length and orientation of neurites from primary rat neurons seeded within the constructs, DRG cells were immunostained with a marker of regenerating neurons. Briefly, constructs were fixed with paraformaldehyde (4% in PBS) overnight and permeabilized in 0.5% w/v Triton X-100 in PBS for 30 min and blocked in 10% goat serum (ThermoFisher, UK) in PBS with 0.5% w/v Triton X-100 for 1 h. Then the primary neuron-seeded constructs were stained with rabbit primary antibody to detect GAP-43 (AB52220, Merck UK, 1:300 in PBS 0.5% Triton X-100) overnight at 4 °C, washed with PBS with 0.5% w/v Triton X-100, and then incubated with Alexa Flour Plus 555 goat antirabbit fluorescent conjugated secondary antibody (A32732, Invitrogen UK) overnight at 4 °C. Constructs were then washed using PBS with 0.5% w/v Triton X-100 and stored in paraformaldehyde in 4 °C until imaged.

### Confocal Microscopy

Confocal microscopy (Zeiss 710 LSM) was used to image the DRG primary neurons within the constructs using a standardized sampling protocol. Images were captured using a × 10 lens, and z-stacks were 100 μm with a step size of 10 μm. Three separate z-stacks at regular intervals along the long axis of the construct were taken per construct, with approximately 20 neurons measured per image. Six independent constructs were imaged per experimental group. Image analysis was conducted using ImageJ by manually tracing the neurites within 2D projections from the 3D z-stacks, to obtain neurite lengths per construct. To obtain an alignment value for the neurons contained within the construct, Volocity (Quorum Technologies Inc, Canada) image analysis software was used. Each neurite was traced in its entirety using Image J to obtain a total length for comparison. For the angle, Volocity software was used (to enable the 3D nature of the neurite orientation to be taken into account), and the overall angle of each neurite was calculated automatically using the built in bearing algorithm.

### Statistical Analysis

Normality was assessed using a Shapiro–Wilk test, then two-way ANOVA analysis was used for neurite extension analysis, and Šídák post hoc tests were used to compare groups. Statistical analysis was performed with GraphPad Prism 9.0.0. Data are represented as mean ± standard deviation (Std. Dev.) or standard error (SEM) if stated otherwise.

## Supplementary Material

Trueman_Aligned_AdvEngMater_2024_Suppl

## Figures and Tables

**Table 1.** Concentrations of hydrogel components. PPy X% refers to the w/w percentage of PPy in relation to the collagen amount in the final construct

Sample name	Collagen (mg per construct)	PPy nanoparticles (mg per construct)
Collagen	1.6	N/A
PPy 2.5%	1.6	0.04
PPy 5%	1.6	0.08

## Data Availability

The data that support the findings of this study are available from the corresponding author upon reasonable request.
