# Supplementary material for: Aligned Bioelectronic Polypyrrole/Collagen Constructs for Peripheral Nerve Interfacing
Source: Adv Eng Mater. Author manuscript; Available in PMC 2025 Mar 1. (PMC11296654; doi:10.1002/adem.202301488)
Supplement: Trueman_Aligned_AdvEngMater_2024_Suppl [file NIHMS2010220-supplement-Trueman_Aligned_AdvEngMater_2024_Suppl.pdf]

Supporting Information

Aligned Bioelectronic Polypyrrole/Collagen Constructs for Peripheral Nerve Interfacing

Ryan P. Trueman [a, b], Owein Guillemot-Legris [a, b], Henry T. Lancashire [c], Abijeet S. Mehta [d], Joshua Tropp [d], Rachel E. Daso [d], Jonathan Rivnay [d, e], Alethea B. Tabor [f], James B. Phillips *[, a, b] & Bob C. Schroeder *[, f]

[a] UCL Centre for Nerve Engineering, University College London, London, United Kingdom

[b] Department of Pharmacology, UCL School of Pharmacy, University College London, London, United Kingdom

[c] Department of Medical Physics and Biomedical Engineering, University College London, London, United Kingdom

[d] Department of Biomedical Engineering, Northwestern University, Evanston, IL, 60208 USA

[e] Simpson Querrey Institute, Northwestern University, Chicago, IL, 60611 USA

[f] Department of Chemistry, University College London, London, United Kingdom

Email : jb.phillips@ucl.ac.uk ; b.c.schroeder@ucl.ac.uk

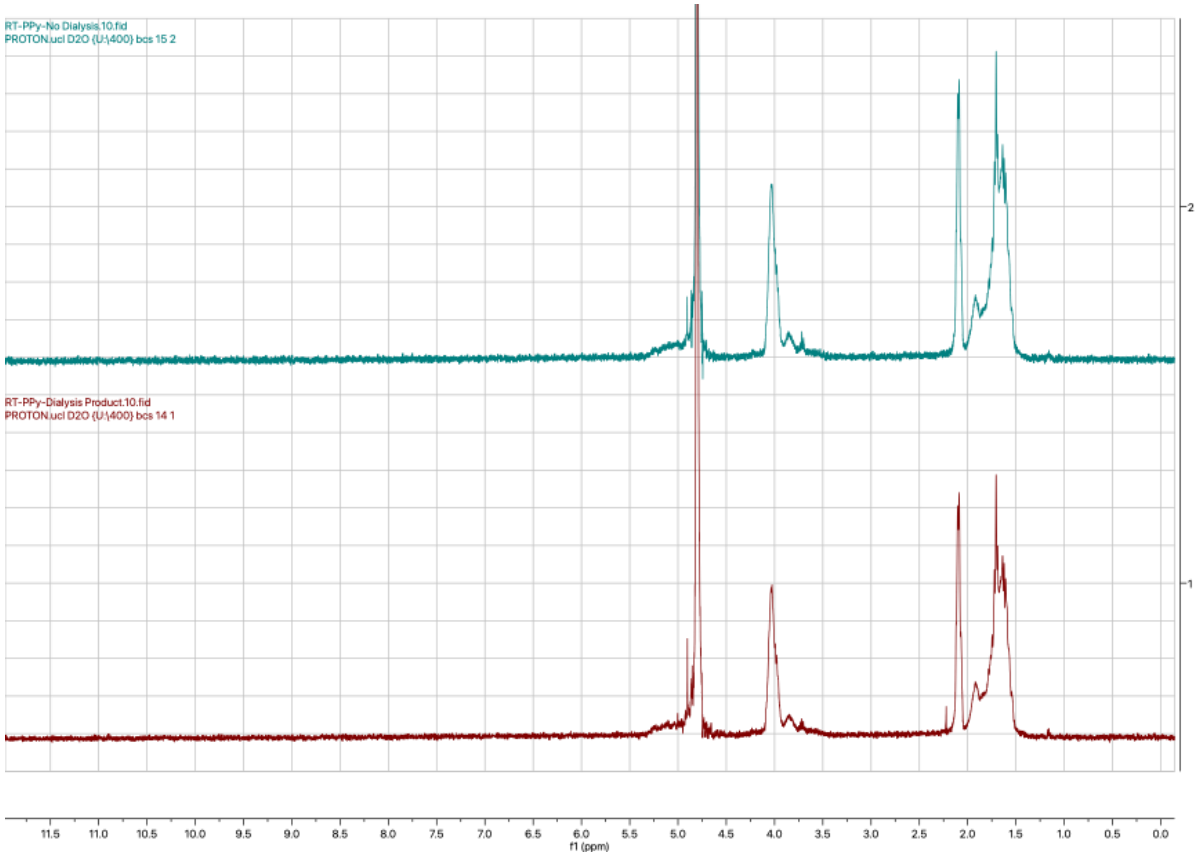

Figure S1 *1H NMR highlighting that the PVA still remains present after purification via dialysis. Blue (top) = 1H NMR before purification. Red (bottom) = After purification via dialysis*

Proton NMR of a dispersion of PPy nanoparticles before and after dialysis (**Figure S1**). The peaks from PVA are still present within the sample, even after purification, indicating the continuous presence of the PVA templating polymer within the nanoparticle synthesis.

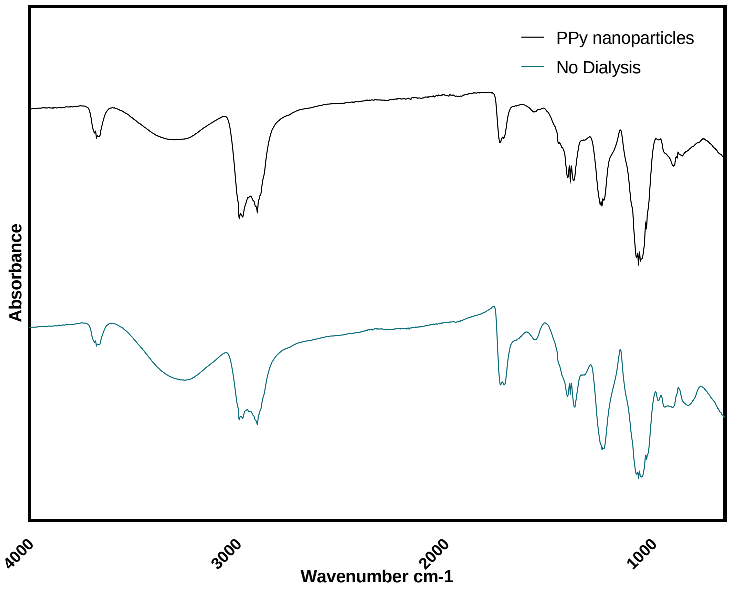

Figure S2 *FTIR of PPy NPs without dialysis (bottom cyan line) and with dialysis (top black line).*

FTIR spectra of the PPy nanoparticles before and after dialysis showed that it was impossible to entirely remove the signals from the PVA (3400 to 3200, and 3000 to 2900 cm-1, O-H and C-H from PVA, respectively) (**Figure S2**). The PVA is hypothesized to stay involved within the templating of the PPy nanoparticles, which would explain the narrow distribution of PPy nanoparticles, as the PVA polymer used was of a specific molecular weight (9000 Da to 10000 Da)

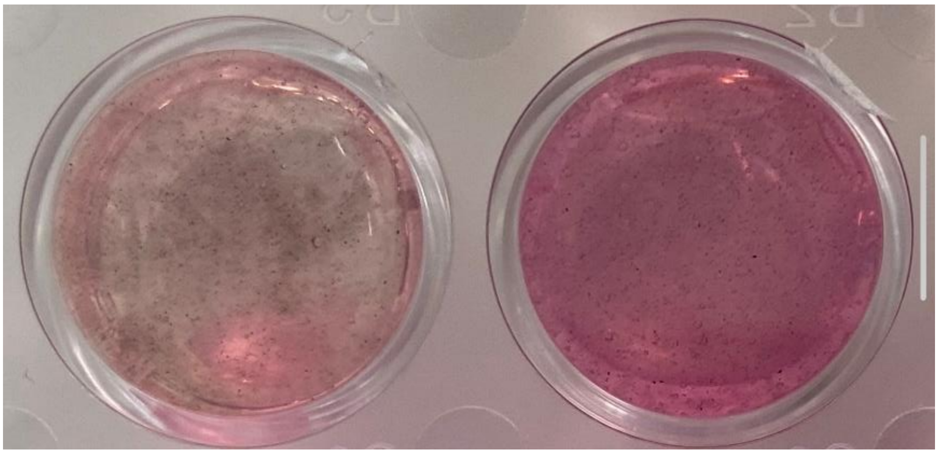

Figure S3 *Photographs of two highly hydrated collagen/PPy composites with a non-size controlled synthesis, displaying aggregation of the PPy. The aggregated PPy particles and a non-homogenous distribution throughout the hydrogel are clearly visible.*

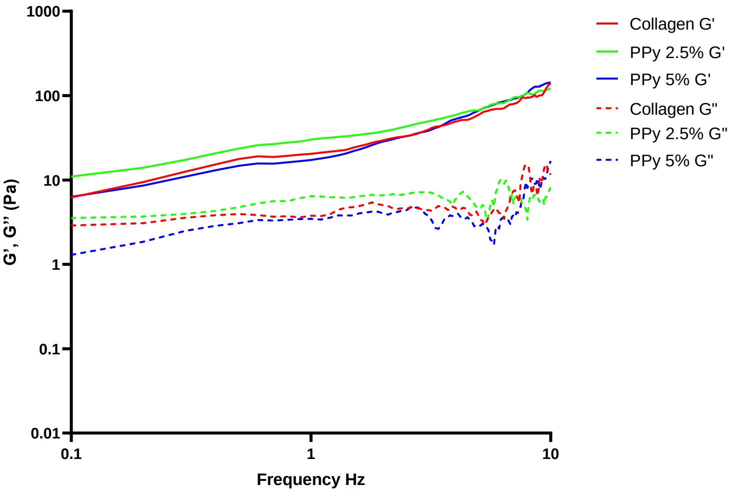

Figure S4 *Rheology of the 2mg/mL collagen and PPy composite hydrogels before processing with GAE. Data presented is mean G' and G'' of 3 experimental repeats between the frequency range of 0.1 to 10 Hz.*

Rheometry was used to investigate the addition of PPy to collagen hydrogels. (**Figure S4**). All materials possess similar rheological features, with G' exceeding G" across the entire frequency range from 0.1 to 10 Hz at a constant strain.

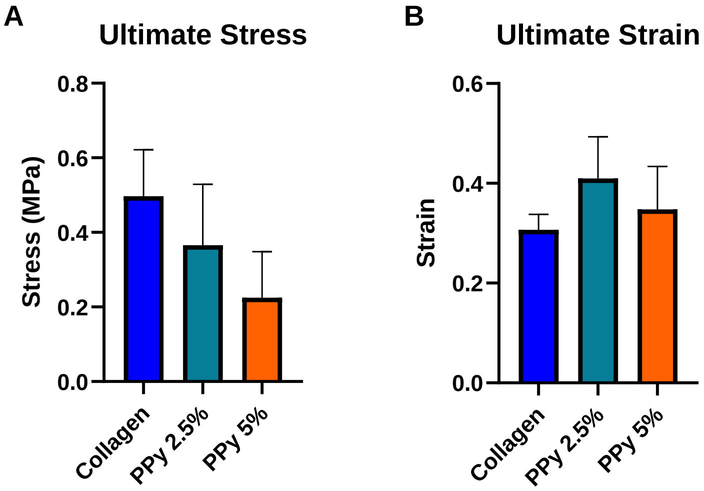

Figure S5 *Ultimate stress and strain from tensile analysis of the constructs, pulled to failure. A) Ultimate stress in MPa for the 3 different constructs. B) Ultimate strain for the 3 different constructs. n=3 independent constructs, with data presented as mean ± S.D.*

Figure S5 represents the ultimate stress and strain on the constructs when a tensile test was performed to failure. Ultimate stress is reduced within the constructs as greater amounts of PPy nanoparticles are added to the collagen prior to GAE. Ultimate strain is not altered to a great extent, with the constructs yielding at similar amounts of strain.

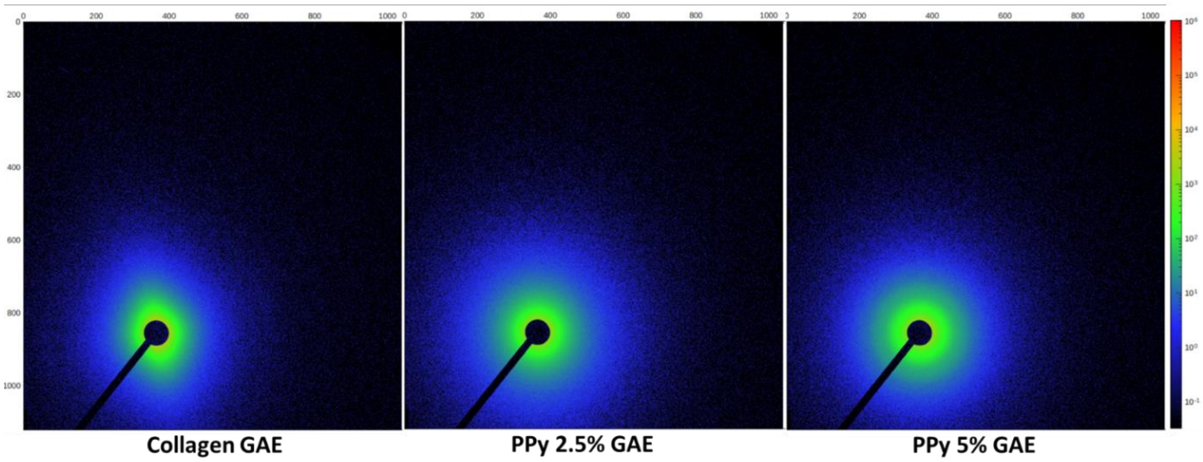

Figure S6 *Small Angle X-Ray Scattering 2D patterns from Collagen, PPy 2.5% and PPy 5% GAE lyophilized GAE constructs*

Figure S6 represents the 2D patterns obtained from small angle x ray scattering (SAXs) performed on the constructs. Anisotropic features are seen for the collagen GAE sample, identified by the ellipsoidal shape, whereas the anisotropy is diminished for samples containing the PPy nanoparticles.

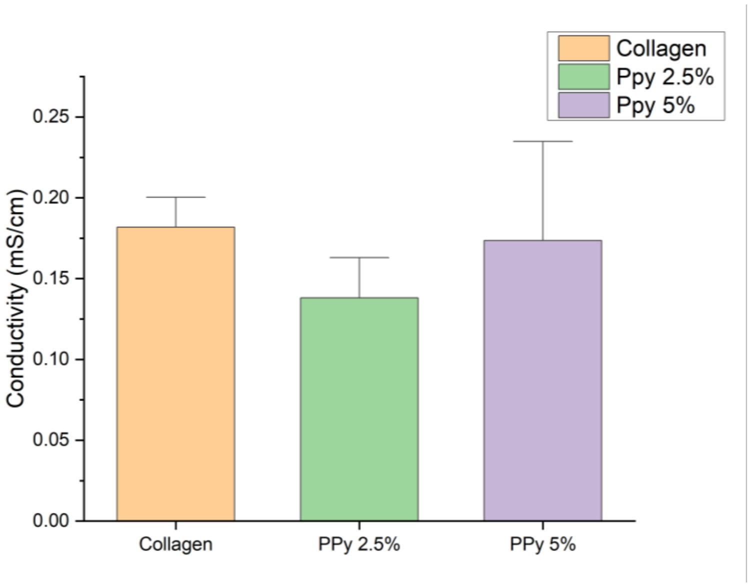

Figure S7 *Conductivity measurements of the highly hydrated gels prior to the GAE process. Measurements were carried out using the same method for the GAE constructs, with EIS and circuit fitting used to generate values for resistance, which were then converted to conductivity using the area of the highly hydrated gels.*

The conductivity of the highly hydrated gels was investigated prior to the application of the GAE process (**Figure S7**). The conductivity of the remains similar between the composite materials.

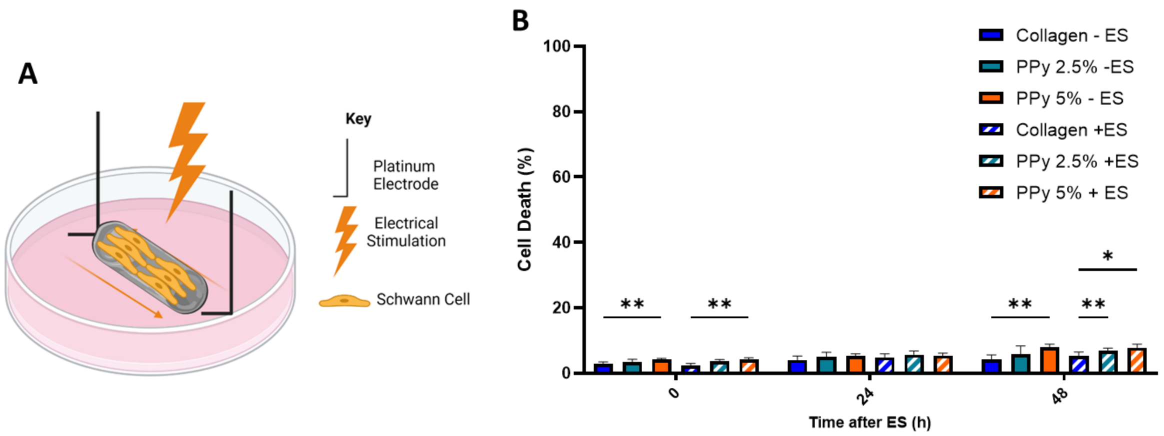

Figure S8 *LDH assay of media containing the Schwann cell loaded constructs, taken at 3 separate time points after electrical stimulation. 2-way ANOVA, with Šídák's post hoc test performed after 2-way ANOVA was deemed significant (p = 0.005). * = p<0.05, ** = p<0.01. Data are presented as means ± SEM. n = 9 separate constructs.*

GAE was successfully employed to create cellular collagen constructs using two separate concentrations of conductive PPy nanoparticle filler. Next, the cell laden constructs were electrically stimulated like graphically represented within **Figure S8**, and cell death was determined using an assay for lactate dehydrogenase (LDH). Cell death remained low within all the samples, with all the groups possessing <10% cell death. However, there was a significant increase in cell death with higher concentrations of PPy (**Figure S8**) for both the electrically stimulated groups and the non-electrically stimulated groups. This highlights that there may be a slight toxic effect, and for this reason we limited the loading of the conductive filler to 5% and did not explore higher concentrations.

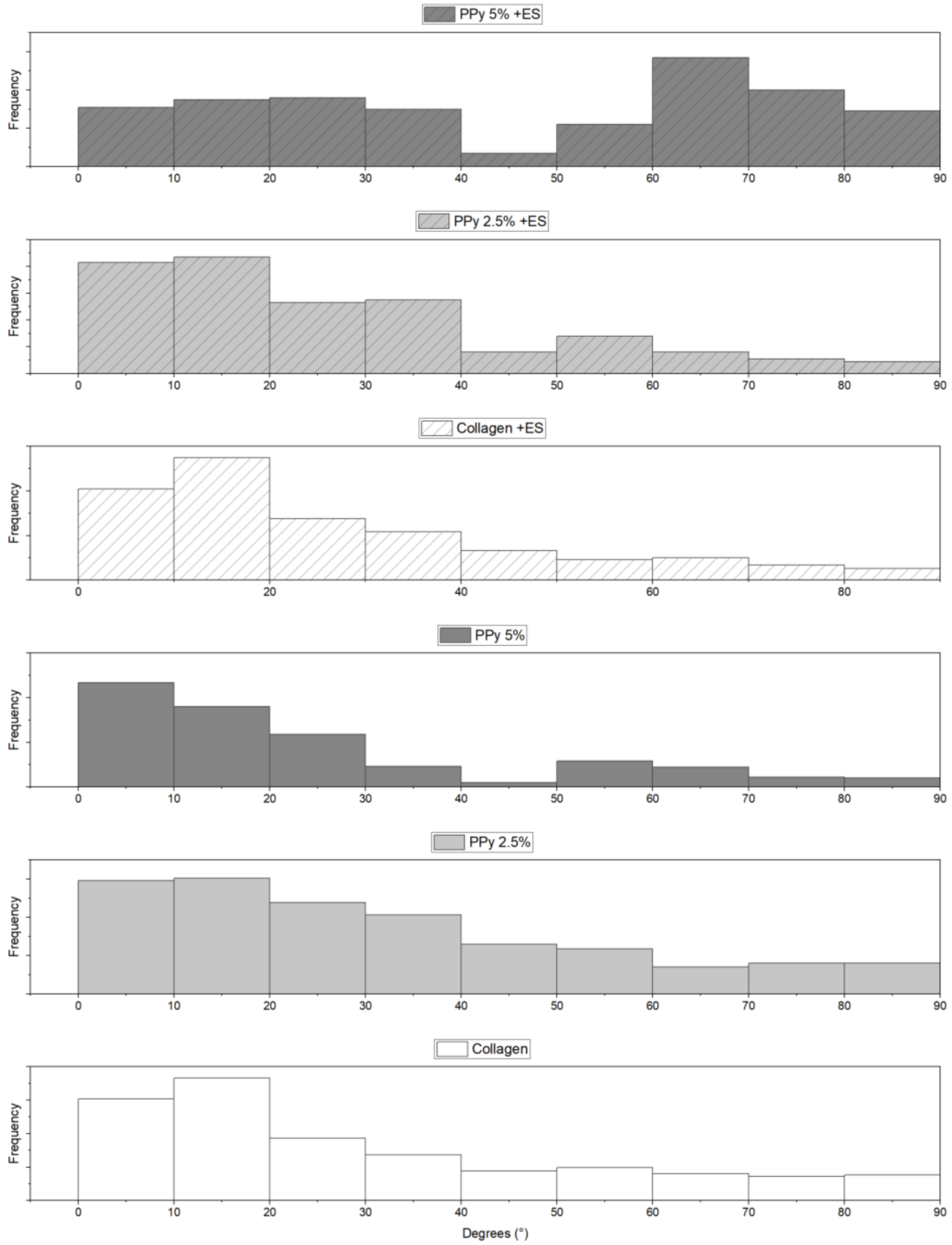

Figure S9 *Histograms taken from the alignment data of the GAP-43 labelled primary neurons within the different composite materials (bottom 3 graphs) and the materials under electrical stimulation (top 3 graphs). Bin width = 10 degrees, with at least 300 neurons per experimental group identified using Volocity software, captured from 6 independent constructs.*

Histograms of the distribution of the neurite extension alignment within the 3D construct were employed to understand the differences in mean alignment between the different materials and electrical stimulation (**Figure S9**). The overall distribution is similar for all the different materials, however there is disrupted alignment within the 5% PPy construct when an electrical stimulation is applied.

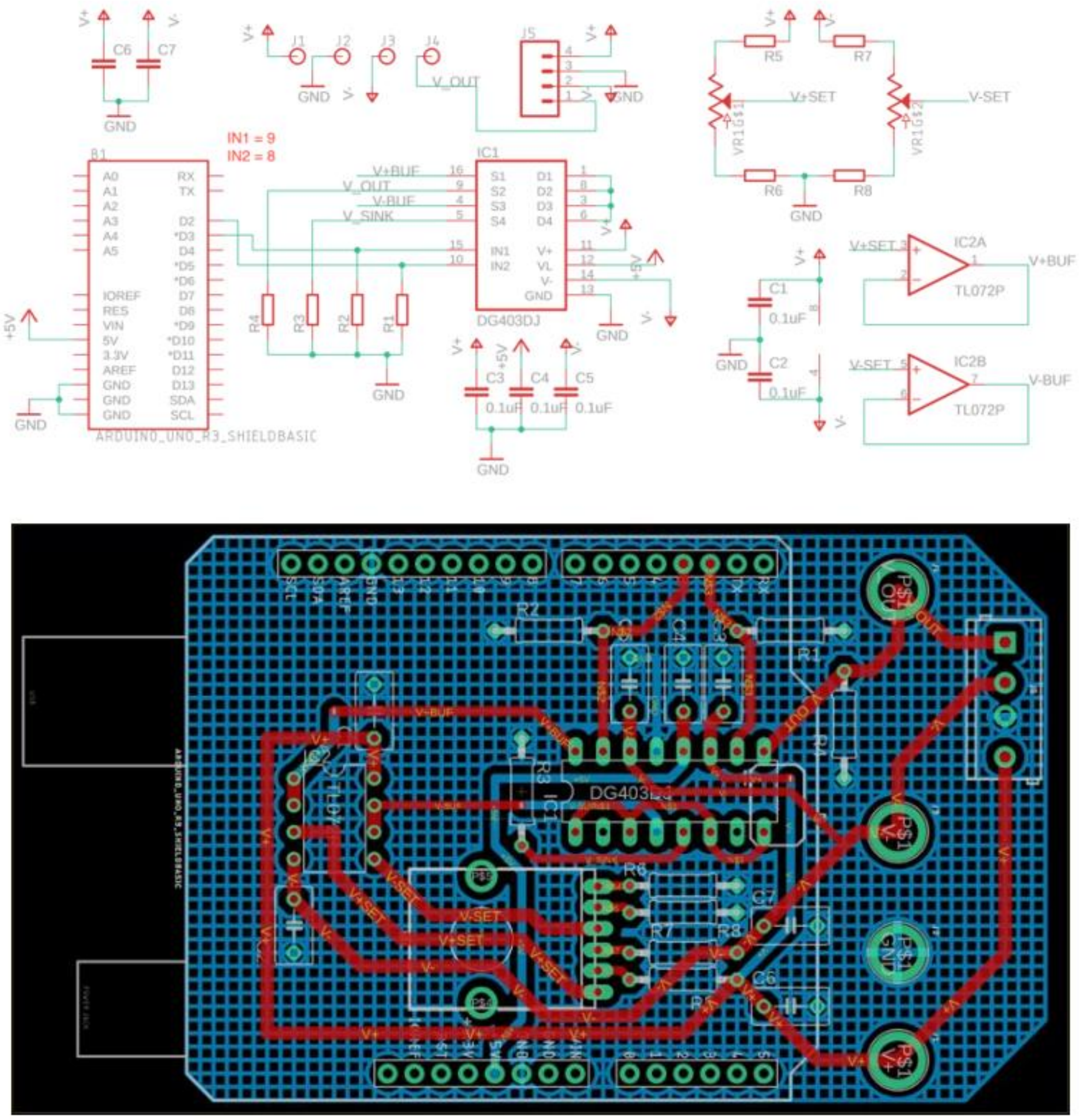

Figure S10 *AutoDesk EAGLE Schematic and Graphical Soldering for the Arduino Waveform generator*

```
/*\
* Ryan Stimulator / Pulse Generator\
*/\
\
unsigned int delaypos = 100; // positive pulse duration in microseconds\
unsigned int delayneg = 100; // negative pulse duration in microseconds\
unsigned int delayip = 50; // interpulse delay in microseconds\
unsigned int delaypp = 50; // pulse-pulse delay in milliseconds. Designed for 20 Hz
stimulation.\
\
void setup() \{\
 // put your setup code here, to run once:\
pinMode(8,OUTPUT); // 8 turns output on\
pinMode(9,OUTPUT); // 9 switches positive (LOW), and negative (HIGH).\
digitalWrite(8,LOW);\
digitalWrite(9,LOW);\
\}\
\
void loop() \{\
 // put your main code here, to run repeatedly:\
\
// Negative Phase\
digitalWrite(9,HIGH); // Setup negative out\
digitalWrite(8,HIGH); // Output on\
delayMicroseconds(delayneg);\
digitalWrite(8,LOW); // Output off\
\
// Interpulse delay\
delayMicroseconds(delayip);\
\
// Positive Phase\
digitalWrite(9,LOW); // Setup positive out\
digitalWrite(8,HIGH); // Output on\
delayMicroseconds(delaypos);\
digitalWrite(8,LOW); // Output off\
\
// Pulse-Pulse delay\
delay(delaypp); // inverse of pulse frequency.\
\
\}\\
```

Figure S11 *Arduino code for the CNEStimulator.*

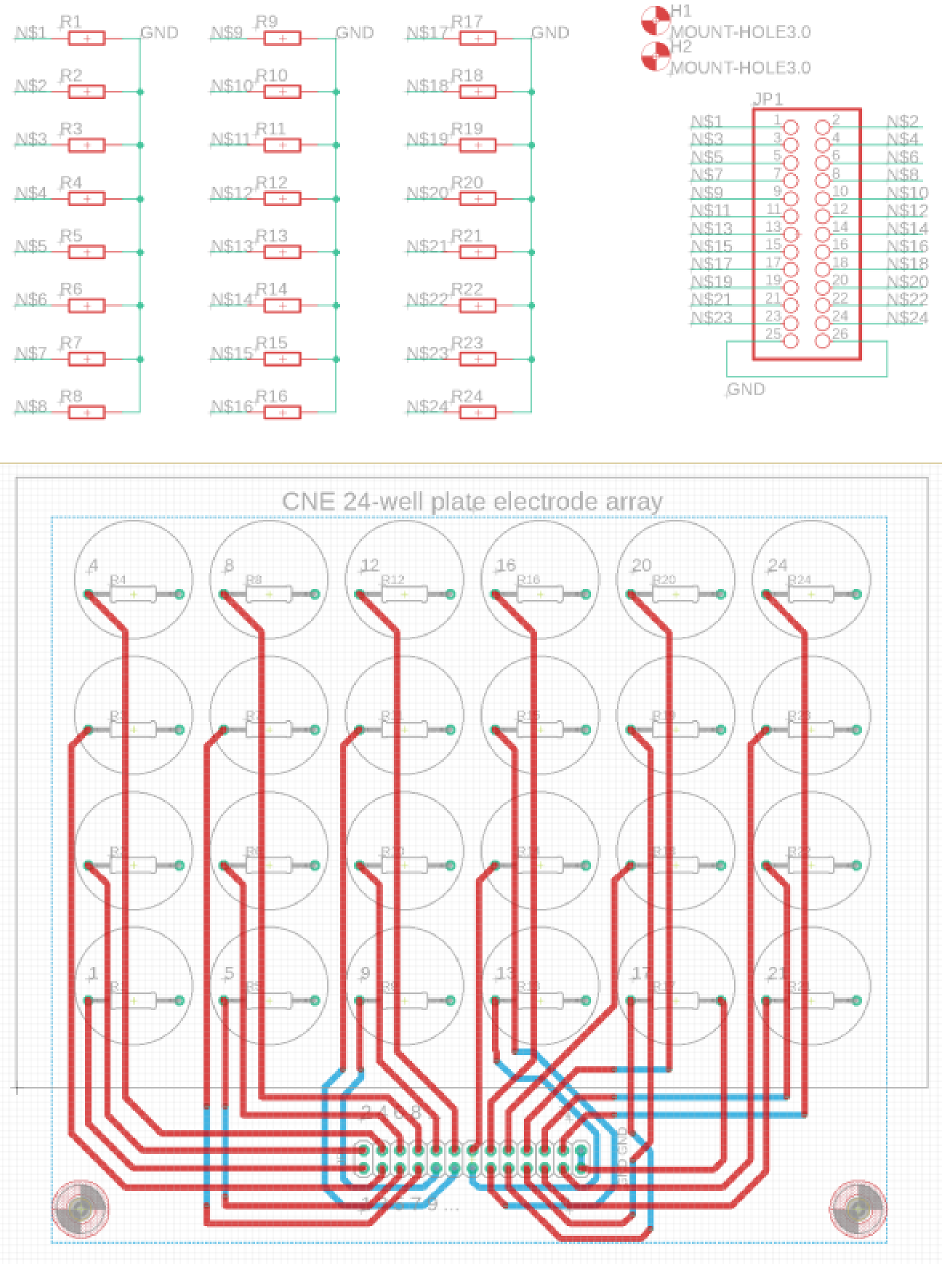

***Figure S12** AutoDesk EAGLE Schematic and Graphical board representation for the CNEStimulator*

Figures S10, S11 & S12 display the overall electrical engineering development during the creation of the CNEStimulator. **Figure S10** displays the AutoDesk Eagle schematics for the design of the Arduino wave form generator responsible for delivering the waveform of electrical stimulation. **Figure S11** highlights the code used in the Arduino integrated developer environment to operate the digital switches present on the Arduino and generate a waveform with both positive and negative components. **Figure S12** displays the Autodesk eagle schematics for the CNEStimulator cell culture lid that integrates the platinum electrodes with the cell culture media and subsequently used within the *in vitro* experiments.
